# Targeting Circadian Protein Rev-erbα to Alleviate Inflammation, Oxidative Stress, and Enhance Functional Recovery Following Brain Trauma

**DOI:** 10.3390/antiox13080901

**Published:** 2024-07-25

**Authors:** Arief Gunawan Darmanto, Jing-Shiun Jan, Ting-Lin Yen, Shin-Wei Huang, Ruei-Dun Teng, Jia-Yi Wang, Rajeev Taliyan, Joen-Rong Sheu, Chih-Hao Yang

**Affiliations:** 1 International Ph.D. Program in Medicine, College of Medicine, Taipei Medical University, Taipei 110, Taiwan; d142109015@tmu.edu.tw (A.G.D.); sheujr@tmu.edu.tw (J.-R.S.); 2 School of Medicine, Universitas Ciputra, Surabaya 60219, Indonesia; 3 Department of Pharmacology, School of Medicine, College of Medicine, Taipei Medical University, No. 250, Wu Hsing St., Taipei 110, Taiwan; d119101004@tmu.edu.tw (J.-S.J.); d119096015@tmu.edu.tw (T.-L.Y.); yd_1124ooo@hotmail.com (S.-W.H.); tang0803@tmu.edu.tw (R.-D.T.); 4 Department of Medical Research, Cathay General Hospital, Taipei 22174, Taiwan; 5 Graduate Institute of Medical Sciences, College of Medicine, Taipei Medical University, Taipei 110, Taiwan; jywang2010@tmu.edu.tw; 6 Department of Neurosurgery, Taipei Medical University Hospital, Taipei 110301, Taiwan; 7 Neuropsychopharmacology Division, Department of Pharmacy, Birla Institute of Technology and Science-Pilani, Pilani Campus, Pilani 333031, Rajasthan, India; taliyanraja@gmail.com

**Keywords:** traumatic brain injury, NR1D1, oxidative stress, inflammation, neuronal cell death, SR9009, SR8278

## Abstract

Traumatic brain injury (TBI) is a significant cause of morbidity and mortality worldwide, and its pathophysiology is characterized by oxidative stress and inflammation. Despite extensive research, effective treatments for TBI remain elusive. Recent studies highlighted the critical interplay between TBI and circadian rhythms, but the detailed regulation remains largely unknown. Motivated by the observed sustained decrease in Rev-erbα after TBI, we aimed to understand the critical role of Rev-erbα in the pathophysiology of TBI and determine its feasibility as a therapeutic target. Using a mouse model of TBI, we observed that TBI significantly downregulates Rev-erbα levels, exacerbating inflammatory and oxidative stress pathways. The regulation of Rev-erbα with either the pharmacological activator or inhibitor bidirectionally modulated inflammatory and oxidative events, which in turn influenced neurobehavioral outcomes, highlighting the protein’s protective role. Mechanistically, Rev-erbα influences the expression of key oxidative stress and inflammatory regulatory genes. A reduction in Rev-erbα following TBI likely contributes to increased oxidative damage and inflammation, creating a detrimental environment for neuronal survival and recovery which could be reversed via the pharmacological activation of Rev-erbα. Our findings highlight the therapeutic potential of targeting Rev-erbα to mitigate TBI-induced damage and improve outcomes, especially in TBI-susceptible populations with disrupted circadian regulation.

## 1. Introduction

Traumatic brain injury (TBI) is a critical public health issue caused by external mechanical forces impacting the head, leading to the impairment of normal brain functioning. Brain trauma can originate from various incidents such as falls, motor vehicle accidents, sports injuries, and assaults, encompassing a broad spectrum of severity from mild concussion to severe brain damage. Epidemiologically, TBI is a leading cause of injury-related death and disability worldwide which results in millions of new cases annually [1,2]. TBI can lead to enduring cognitive deficits, physical disabilities, and mental health conditions such as depression and anxiety, significantly diminishing the quality of life for individuals suffering with TBI [3,4] and potentially causing considerable strain on economic and healthcare systems [5]. Consequently, there is an urgent need for effective treatments to mitigate these impacts and improve outcomes for TBI patients.

Oxidative stress and inflammation play crucial roles in the pathology of TBI, causing immediate and long-term neuronal damage. After the initial injury, the overproduction of reactive oxygen and nitrogen species (ROS and RNS, respectively) leads to oxidative damage to DNA, proteins, and lipids, disrupting brain structure and cellular homeostasis [6,7]. Meanwhile, the induction of inflammatory events further exacerbates the damage through the release of pro-inflammatory cytokines like IL-1β and TNF-α, which activate microglia and astrocytes. This inflammatory response establishes a feedback loop, with ROS and cytokines inducing each other’s release, ultimately leading to extensive tissue impairment and cell death [6,8]. Effective therapeutics targeting oxidative stress and inflammation could be promising treatments to mitigate the deleterious impacts of TBI.

As the circadian system regulates a variety of physiological processes, its disruption is increasingly recognized as a significant factor in the pathology of TBI. Accumulating evidence indicates alterations in circadian rhythms following brain trauma impact sleep–wake cycles [9], hormone release [10], and cognitive function [11]. Interestingly, disrupted circadian rhythms following TBI are associated with impaired neurogenesis, exacerbated inflammation, and increased oxidative stress, all of which hinder recovery and cause a long-term interruption in brain function [11,12,13]. Moreover, studies have indicated that restoring circadian rhythm might alleviate neuroinflammation and improve functional outcomes in TBI [14], highlighting the therapeutic potential of targeting the circadian system in TBI treatment.

NR1D1 (nuclear receptor subfamily 1, group D, member 1), also known as Rev-erbα, functions as a transcriptional regulator that controls multiple physiological processes, including circadian rhythm regulation, autophagy, metabolism, and inflammation [15,16]. Recent research has highlighted the role of Rev-erbα in modulating oxidative stress and inflammatory events. It plays a protective role in the vasculature by modulating inflammation and oxidative stress that stabilizes fragile plaques, thereby preventing thrombosis and cardiovascular disorders [17]. In addition, Rev-erbα regulates ROS generation and NRF2-associated enzymes for cytoprotection and reduces synovial inflammation and bone destruction in rheumatoid arthritis [18]. Its dysregulation is linked to various diseases, underscoring its importance in cellular homeostasis [19]. As a core circadian regulator, Rev-erbα might serve as a key mediator of the crosstalk between oxidative stress and inflammation [17,20,21], influencing the progression and recovery from brain trauma. However, the potential role of Rev-erbα in modulating the severity of TBI has not yet been evaluated.

## 2. Materials and Methods

### 2.1. Animals

Adult C57BL/6 mice (male, 8 weeks old, 25–30 g) used for the current study were obtained from BioLASCO (Taipei, Taiwan). The experimental protocols were reviewed and approved by the Institutional Animal Care and Use Committee of Taipei Medical University with the approval number as LAC2022-0480. Prior to the experiments, all animals were first confirmed to be in normal condition, pathogen-free, and without any neurological impairments. After we received the mice, they were allowed to undergo a 7-day acclimatization period upon arrival. All animals were housed in a conventional pathogen-free facility under standard temperature and humidity conditions, with a 12 h light/dark cycle (lights on 0700; lights off 1900).

### 2.2. The Mouse Model for Traumatic Brain Injury

A controlled cortical impact (CCI) device was employed to induce traumatic brain injury, following a previously established protocol with minor adjustments [22]. Mice were initially anesthetized using 4% isoflurane for induction and then placed on a stereotaxic frame. Then, the skin was retracted to expose the skull, and the procedure of craniotomy was performed on the right cerebral hemisphere, creating a circular opening with a diameter of 3.5 mm. This opening was positioned at the center of the circle, located two mm lateral to the sagittal suture and 2 mm posterior to the bregma, exposing the dura mater.

The exposed dura mater was then impacted with a 3 mm impactor at a velocity of 5 m/s with the depth of 1 mm, and a dwell time of 300 ms to induce the traumatic damage to the mouse brain. Following the CCI procedure, the scalp incision was stitched, and the mouse was allowed to recover on a heating pad before being returned to its home cage.

### 2.3. Experimental Designs

A cohort of animals was randomly distributed into two sets of experiments, each containing four groups based on treatment with either the Rev-erbα antagonist SR8278 or the Rev-erbα agonist SR9009. Experiment 1 with Rev-erbα antagonist treatment includes sham/vehicle that received vehicle injection, TBI/vehicle mice that received vehicle injection after TBI, sham/SR8278 that received SR8278 injection and TBI/SR8278 that received SR8278 following CCI surgery. Experiment 2 with Rev-erbα agonist treatment includes sham/vehicle that received vehicle injection, TBI/vehicle mice that received vehicle injection after TBI, sham/SR9009 that received SR9009 injection and TBI/SR9009 that received SR9009 following CCI surgery.

For vehicle and drug treatments, the groups were administered isovolumetric solvent (1% DMSO, intraperitoneal injection (i.p.)), SR9009 (50 mg/kg, i.p.), or SR8278 (25 mg/kg, i.p.) at the time points of one, twenty-four and forty-eight hours after the CCI challenge. The doses of SR9009 (50 mg/kg, i.p.) and SR8278 (25 mg/kg, i.p.) used in our study are based on previous publications and studies that have demonstrated their efficacy and safety in modulating Rev-erbα activity [23,24]. For behavioral experiments, mice from each group were subjected to pre-test evaluations of their neurological severity score (NSS), grip strength, novel object recognition, and Y-maze performance three days before CCI challenge. Post-test measurements were conducted at one, three and seven days after TBI.

Ten mice for each experimental group were used for the behavioral experiments, four mice for each experimental group were used for the immunostaining, and five mice for each experimental group were used for the biochemical analyses such as Western blot and quantitative real-time PCR analyses. In total, 160 mice were used in the current study.

### 2.4. Novel Object Recognition Task

In order to evaluate recognition memory mediated by the hippocampus, we implemented the novel location recognition test [25]. Basically, the test was conducted in a white-colored box measuring 50 × 50 × 50 cm^3^, positioned in the center of a dimly lit room. During the habituation session, the animals were given twenty minutes to freely explore the entire area of the empty box without any testing objects. Various visual cues were marked on the walls of the testing apparatus to provide contextual guidance for the mouse.

Following the twenty minutes of habituation session, the mice were returned to the testing apparatus one hour later, where they were allowed to explore two identical objects placed next to each other for another 10 min. After this training session, the mice were placed back in their home cages. One hour later, a testing session was conducted in which one of the objects was relocated to the other position of the testing chamber. During the test, the mouse was allowed to explore the two objects for a period of 10 min. A digital video camera recorded the time the mouse spent exploring each object. The analysis was performed using Ethovision XT (Noldus), a behavioral tracking system, to score the preference for the newly located object. We calculated a discrimination index using the formula [time spent on the newly located object/(time spent on the newly located object + time spent on another object)] to measure the mouse’s hippocampus-dependent cognitive performance.

### 2.5. Neurological Severity Score (NSS)

For the understanding of sensorimotor coordination following CCI challenge for the mice, all the mice underwent a neurological examination before and at different time points after CCI challenge. The assessment of neurological severity score involved evaluating motor function performance through muscle movement, visual and tactile responses, proprioception, balance, and reflexes. The NSS score was determined using an 18-point sliding scale, where a score of zero indicates normal function and a score of 18 indicates the maximum neurological impairment [26]. Each task or response was scored based on the mouse’s ability to perform it, the total score was the sum of all individual scores, with a maximum possible score of 18 indicating the severe neurological impairment.

### 2.6. Grip Strength Test

Grip strength tests were conducted to assess neuromuscular strength after the CCI challenge by using a grip strength meter (UGO Basile, Varese, Italy). Briefly, mice were placed on the grid horizontally, with their front paws attached to the sensor grip at the same height as the hind paws. Once the mice comfortably gripped the sensor grip, they were pulled back by their tails at a constant speed until they released the sensor grip. The peak tension exerted by the front paws was recorded as gram-force (gf). Each mouse underwent three tests, and the average value was measured before and at three and seven days after TBI for statistical analysis.

### 2.7. Y-Maze Testing

Evaluation of spatial working memory was conducted using the spontaneous alternation Y-maze test, which basically relied on the rodents’ tendency to investigate new locations instead of familiar ones [27]. We utilized a Y-shaped maze with different visual cues attached to the walls of each of the three arms as the experimental testing setup. The whole test was set up and took place in a dimly lit room. At the beginning of the test, the mice were positioned at the center of the Y-shaped maze and allowed to explore the three arms freely for 10 min. A video recording system captured and analyzed the sequential order of each visit to the three different arms to investigate their spontaneous alternation behavior during the exploration period.

An arm visit was defined when the center of the mouse’s body entered a specific arm. A consecutive entry into three different arms was defined as an alternation. The percentage of alternation was calculated by dividing the total number of alternations by the maximum possible alternations (total number of arms entered—2), and then multiplying the result by 100% and reflect the performance in spatial working memory for the mouse.

### 2.8. Histological and Immunofluorescence Analyses

**Figure 1:**
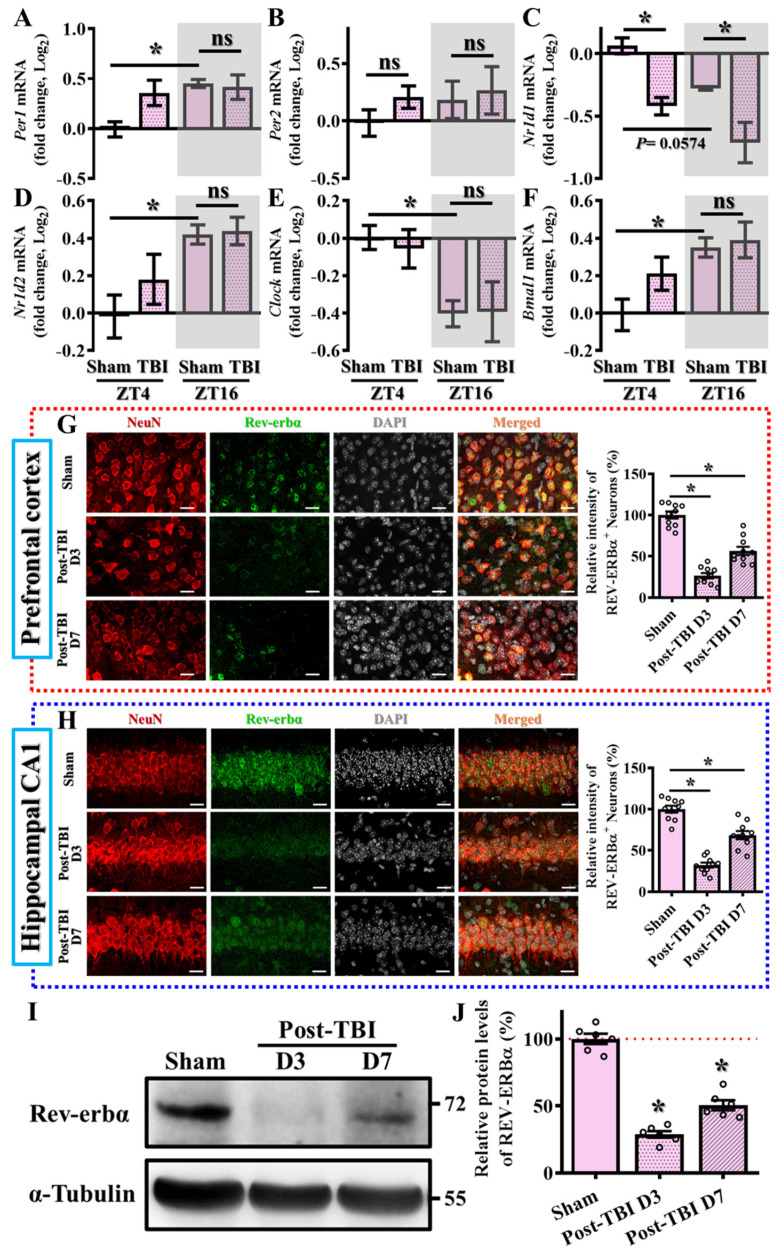
Alteration in the levels of circadian genes following TBI. (**A**–**F**) Quantification of the fold changes in gene expression levels of *Per1*, *Per2*, *Nr1d1*, *Nr1d2*, *Clock*, and *Bmal1* between different experimental groups at ZT4 and ZT16. *n* = 5 mice. ZT4 (Zeitgeber time) refers to the time 4 h after lights are turned on, and ZT16 represents the time 4 h after lights off. (**G**) Representative images and quantitative analyses for the expression of Rev-erbα-positive (green), neuron (NeuN, red) and DAPI (grey) in the prefrontal cortex between different experimental groups (scale = 20 μm). *n* = 10 images from 4 mice. (**H**) Representative images and quantitative analyses for the expression of Rev-erbα-positive (green), neuron (NeuN, red) and DAPI (grey) in the hippocampal CA1 regions between different experimental groups (scale = 20 μm). *n* = 10 images from 4 mice. (**I**,**J**) Representative Western blots and quantitative analyses for the relative protein expression levels of Rev-erbα between different experimental groups. All the results were presented as mean ± SEM and analyzed by a one-way ANOVA followed by Bonferroni’s post hoc analysis. * *p* < 0.05; ns represents not significant.

For the preparation of brain sections for the experiments of immunofluorescent staining, mice were euthanized by deep anesthesia at three days after the procedure of sham operation or CCI challenge. And then the mouse was transcardially perfused with phosphate-buffered saline (PBS) followed by 4% paraformaldehyde (PFA) solution (Sigma-Aldrich, Merck, Germany). Following perfusion, the brains from different experimental groups were extracted, fixed with 4% PFA overnight at 4 °C, and then placed in 30% sucrose at 4 °C for three days until they completely sank. Using a sliding microtome (Leica SM2010R, Germany), the brains were sectioned to a thickness of 50 µm and subjected to immunofluorescence staining. All mice in the current study, excluding those used for experiments in Figure 1, were sacrificed at ZT10 (5 PM) to avoid the influence of circadian differences in gene expression levels or cellular events.

For the immunofluorescence staining, the brain tissue sections were first immersed in donkey serum and Triton X-100 for an hour, and then exposed to a mixture of primary antibodies overnight at 4 °C. The antibodies used and their concentrations were as follows: NeuN (Sigma-Aldrich, Darmstadt, Germany, ABN91, 1:1000), NR1D1 (Abcam, Cambridge, UK, ab174309, 1:500), Iba1 (Wako Chemicals, Osaka, Japan, 019-19741, 1:1000), GFAP (Abcam, ab4674, 1:1000), 4-Hydroxynonenal (4-HNE) (Abcam, ab46545, 1:500), 8-oxo-2’-deoxyguanosine (8-oxo-dG) (JaICA, Tokyo, Japan, MOG-100P, 1:200), Superoxide Dismutase 1 (SOD1) (GeneTex, Irvine, CA, USA, GTX100554-GTX, 1:500). The tissue sections were then exposed to the secondary antibodies with CF488A, CF568, or CF633 (Biotium, Fremont, CA, USA) overnight at 4 °C. Following three rounds of washes with Tween 20 in PBS, sections were sealed and cover-slipped with ProLongTM Glass Antifade Mountant solution. Imaging of immunofluorescent-stained brain sections was performed using a Leica STELLARIS 8 (Leica Microsystems, Wetzlar, Germany) with 40× magnification. The prefrontal cortical areas or hippocampal CA1 regions were manually counted in three selected areas of 50 × 50 µm^2^ per image and presented as the number of positive neurons per mm^2^.

### 2.9. TUNEL Staining for the Evaluation of Apoptotic Cell Death

Brain sections from different experimental groups were prepared and mounted on slide glasses and then subjected to antigen retrieval using Proteinase K solution for a period of fifteen minutes. The sections were then incubated with a blocking agent, followed by primary NeuN antibody (Merck, Darmstadt, Germany) and anti-chicken 633 secondary antibody (Biotium, Fremont, CA, USA) as per the previously described protocols. After the staining with NeuN signal, the brain sections were washed with PBS and subjected to the terminal deoxynucleotidyl transferase dUTP nick end labeling (TUNEL) staining using the Click-iT Plus TUNEL Assay Kit (C10617, Thermo Fisher Scientific, Waltham, MA, USA) following the manufacturer’s instructions. After the staining of the TUNEL signal, brain sections were sealed and cover-slipped with ProLongTM Glass Antifade Mountant solution.

Imaging of the immunofluorescent signals was performed using a Leica STELLARIS 8 (Leica Microsystems, Wetzlar, Germany) with 40× magnification. The prefrontal cortical areas or hippocampal CA1 regions were manually counted in three selected areas of 50 × 50 µm^2^ per image, and the results were eventually presented as the number of TUNEL-positive neurons per mm^2^.

### 2.10. Real-Time PCR Measurement

After the CCI challenge, mice from different experimental groups were euthanized three days after the procedure of sham operation or CCI surgery. All mice in the current study, excluding those used for experiments in Figure 1, were sacrificed at ZT10 (5 PM) to avoid the influence of circadian alterations in gene expression levels. The mice were anesthetized and perfused with cold PBS solution. Subsequently, the cortex and hippocampal tissues were promptly collected, rapidly frozen in liquid nitrogen, and homogenized. Total RNA was extracted using NucleoSpin RNA (Macherey-Nagel, Düren, Germany). Following the measurement of total RNA concentration, 2 µg of total RNA was reverse transcribed into cDNA using the SuperScript™ IV First-Strand Synthesis System (Thermo Fisher Scientific). The cDNA samples from each experimental group were then quantified by using the QuantiNova PCR Kits (Qiagen, Hilden, Germany) with the Real-Time PCR System (Applied Biosystems™, Thermo Fisher Scientific). The sequences of the primer pairs used for amplification of each target are listed in Table 1.

### 2.11. Western Blotting

For the biochemical analyses of changes in protein expression levels of Rev-erbα in the hippocampus following TBI, coronal brain sections with the thickness of 1 mm containing the hippocampal CA1 regions were surgically dissected under a dissecting microscope. The tissues from different groups were first homogenized and sonicated in an ice-cold Tris-HCl lysis buffer solution (pH 7.4) containing a mixture of protein phosphatase and proteinase inhibitors and then centrifuged at 12,000× *g* for twenty minutes at 4 °C. Samples of equal amounts of proteins were subjected to 10% SDS-PAGE, and the proteins were then electro-transferred to PVDF membranes using a Bio-Rad semi-dry transfer unit (Hercules, CA, USA). The membranes were blocked with 5% (*w*/*v*) non-fat milk in TBST (10 mM Tris-base, 100 mM NaCl, and 0.02% Tween 20) for an hour and then probed with primary antibodies overnight at 4 °C, specifically Rev-erbα (Abcam, ab174309, 1:500) and α-Tubulin (Sigma-Aldrich, T6074, 1:10,000). After extensive washing with TBST, the membranes were incubated with the appropriate HRP-linked secondary antibody at a concentration of 1: 1000. The immunoreactive bands were visualized with enhanced chemiluminescent reagents (ECL, Amersham, UK) using a scientific imaging system (Biospectrum AC System, UVP, Upland, CA, USA) and analyzed with Image J (Version 1.54, NIH, New York, NY, USA) software plugins.

### 2.12. Statistical Analysis

All the results are presented as the mean ± SEM. The one-way analysis of variance (ANOVA) was conducted to compare values among different groups with more than two experimental groups and then Bonferroni’s post hoc analyses were conducted to assess for significant difference between any of the isolated experimental groups. Pearson’s correlation analysis was carried out to investigate the correlation between two parameters between individual animals. A probability value of *p* < 0.05 was considered to indicate statistical significance for the analyses. All statistical analyses were then illustrated and presented using the Prism 8 software (GraphPad, San Diego, CA, USA).

## 3. Results

### 3.1. Alteration in the Levels of Circadian Genes Following TBI

Given the well-known interplay between circadian regulation and TBI functional outcomes, we wanted to first investigate alterations in key circadian regulators in the brain following TBI. By using a well-established mouse model for TBI, the controlled cortical impact (CCI), we first evaluated TBI-induced changes in the expression levels of circadian regulators, including Per1, Per2, Nr1d1, Nr1d2, Clock, and Bmal1, at two circadian time points (ZT4 and ZT16) three days post-TBI. Our findings revealed a circadian pattern in the expression of these regulators (Figure 1A–F), with some showing significant alterations after the challenge of TBI. Notably, there is a significant decrease in Nr1d1 mRNA levels at both ZT4 and ZT16 following TBI (Figure 1C; sham/ZT4, 0.064 ± 0.06 vs. TBI/ZT4, −0.421 ± 0.07, *p* < 0.05; sham/ZT16, −0.278 ± 0.014 vs. TBI/ZT16, −0.712 ± 0.162, *p* < 0.05).

Additionally, we also confirmed the protein expression levels of Rev-erbα via immunofluorescent staining. By preparing brain sections from sham-operated or TBI mice at three and seven days after TBI, we found there was a significant decrease in the protein expression levels of Rev-erbα in both the prefrontal cortex and hippocampal CA1 regions (the areas implicated in cognitive function changes following brain trauma) at three days post-TBI (Figure 1G,H). Although there was a gradual recovery of Rev-erbα protein levels at seven days post-TBI, they remained significantly decreased compared to the sham-operated group of animals (Figure 1G,H) indicating a sustained alteration in the expression of the key circadian regulator Nr1d1 following TBI. Similar results demonstrating the decrease in Rev-erbα protein levels post-TBI were also confirmed by Western blotting (Figure 1I,J).

### 3.2. Levels of Nr1d1 Correlate to TBI Phenotypes

**Figure 2:**
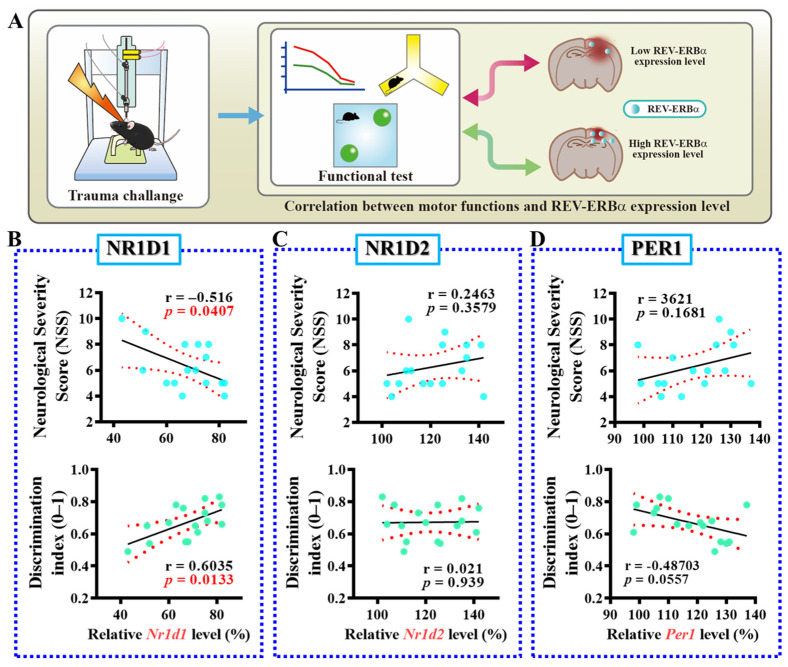
Correlation of the levels of circadian genes with TBI-related phenotypes. (**A**) The illustrating image demonstrates the experimental procedures for the correlation analyses to examine the relationship between individual expression levels of circadian genes with their behavioral outcomes post-TBI. (**B**) Pearson’s correlation plots for the relative *Nr1d1* levels with individual motor or cognitive function after TBI. (**C**) Pearson’s correlation plots for the relative *Nr1d2* levels with individual motor or cognitive function after TBI. (**D**) Pearson’s correlation plots for the relative *Per1* levels with individual motor or cognitive function after TBI. *n* = 15 mice.

In order to understand if the observed alteration in the expression levels of Nr1d1 might functionally link to the outcomes following TBI, we then conducted Pearson correlation analyses to examine the relationship between individual expression levels of Nr1d1 with their behavioral outcomes post-TBI. For the functional behavioral outcomes, neurological severity scores (NSS), which assess motor, sensory, reflex, and balancing performance, were evaluated to reflect the proper behavioral performance in motor functioning. In addition, the novel object location task was used for the evaluation of hippocampus-dependent cognitive performance (Figure 2A). We found that the expression levels of Nr1d1 were negatively correlated with higher NSS scores, indicating poor motor performance, and positively correlated with a higher discrimination index in the novel object location task, indicating improved cognitive performance (Figure 2B). These results indicate a possible protective role of Nr1d1 in the pathology of TBI. In contrast, there was no significant correlation between Nr1d2 or Per1 expression levels and motor or cognitive performance post-TBI (Figure 2C,D). These findings strongly indicate that decreased Nr1d1 after traumatic brain injury may contribute to TBI-induced deleterious impacts, and pharmacological agents targeting its function could provide therapeutic benefits.

### 3.3. Pharmacological Inhibition of Rev-Erbα Slows Down Recovery from TBI

**Figure 3:**
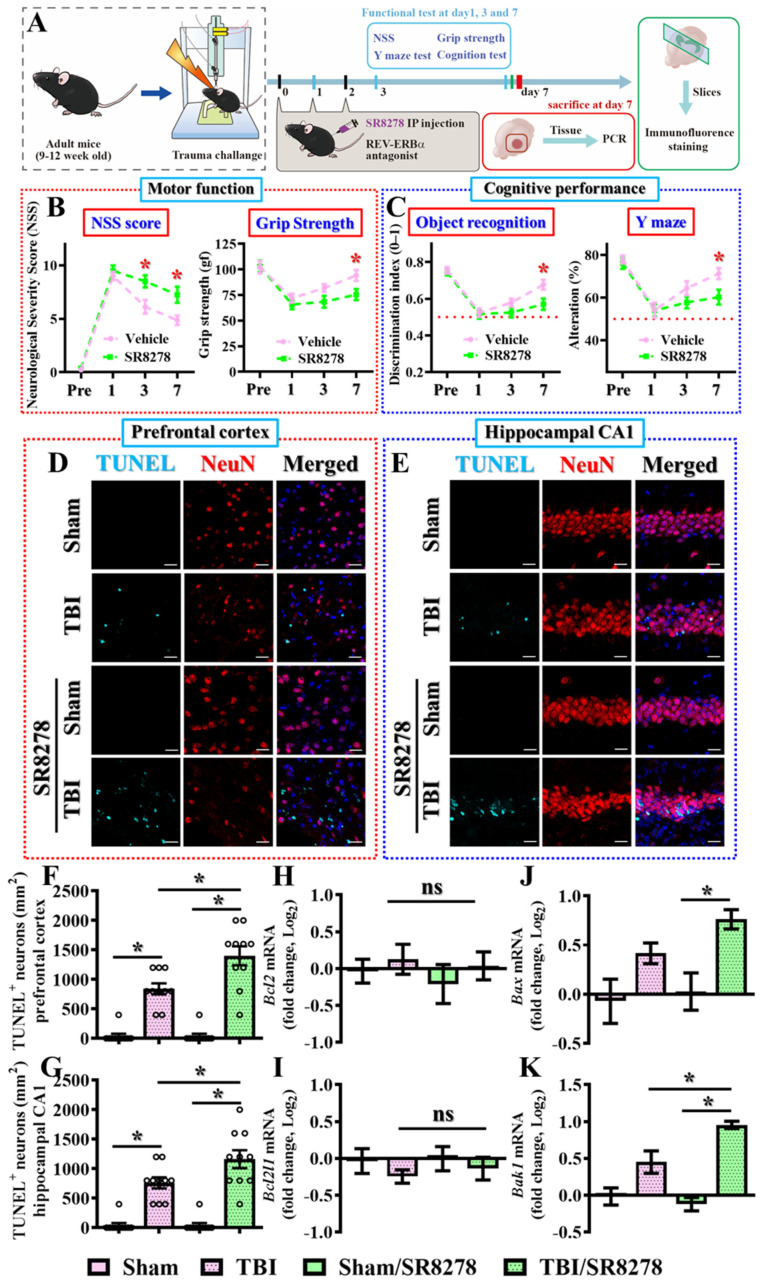
Pharmacological inhibition of Rev-erbα slows down recovery from TBI. (**A**) The illustrating image demonstrates the procedures for the experimental designs. (**B**) Behavioral performance for the evaluation of motor function based on neurological severity scores and grip strength at different time points after TBI challenge. Data are presented as mean ± SEM. *n* = 10 mice. (**C**) Cognitive performance was evaluated based on the object recognition and Y-maze task at different time points after TBI challenge. Data are presented as mean ± SEM. *n* = 10 mice. (**D**) Representative images for the TUNEL-positive (cyan), neuron (NeuN, red) and DAPI (blue) signals in the prefrontal cortex from different experimental groups (scale = 20 μm). (**E**) Representative images for the TUNEL-positive (cyan), neuron (NeuN, red) and DAPI (blue) in the hippocampal CA1 regions from different experimental groups (scale = 20 μm). (**F**) Quantification of the number of TUNEL-positive neuronal cells in the prefrontal cortex. *n* = 10 images from 4 mice. (**G**) Quantification of the number of TUNEL-positive neuronal cells in the hippocampal CA1 regions from different experimental groups. *n* = 10 images from 4 mice. (**H**–**K**) Quantification of the fold changes in gene expression levels of *Bcl2*, *Bcl2l1*, *Bax* and *Bak1* in the hippocampus between different experimental groups. *n* = 5 mice. Data are presented as mean ± SEM and then analyzed by the one-way ANOVA test followed by Bonferroni’s post hoc analysis. * *p* < 0.05; ns represents not significant.

To understand the possible role of Rev-erbα in the modulation of an individual’s functional recovery from brain trauma, we first designed a loss-of-function experiment to suppress Rev-erbα activity and investigate its effects on TBI recovery. Since the prolonged suppression of core circadian genes through either genetic knockout or silencing may disrupt proper circadian regulation, we opted for a temporal suppression of Rev-erbα activity by using the pharmacological antagonist SR8278. We first confirmed that the temporal inhibition of Rev-erbα activity did not significantly alter diurnal differences in locomotor activity at seven days after intraperitoneal injection of SR8278. Additionally, the functional inhibition of Rev-erbα by SR8278 was confirmed by a significant increase in the mRNA levels of Bmal1, which has long been found as a direct target transcriptionally suppressed by Rev-erbα (Figure S1), without significant alterations in the mRNA levels of Nr1d1 and Per1. Experimentally, adult mice aged between nine to twelve weeks of age received 25 mg/kg SR8278 intraperitoneally at one, twenty-four, and forty-eight hours after CCI challenge and were evaluated for their behavioral performance in motor and cognitive function at one, three, and seven days following brain trauma (Figure 3A). We found that the CCI-induced decrease in behavioral performance gradually recovered after three and seven days following TBI in the vehicle-injected control animals (Figure 3B,C), and the pharmacological inhibition of Rev-erbα significantly slowed down the functional recovery of behavioral performance in motor function, as reflected by higher NSS scores and poorer grip strength compared to the vehicle-injected group of animals (Figure 3B). Meanwhile, the injection of SR8278 also blocked the recovery of cognitive functions following TBI, as indicated by decreased discriminative capability in the novel object location task and decreased alternation in new arm selection in the spontaneous alternation Y-maze task (Figure 3C).

The observation of an impairment in functional recovery from TBI in the animals with the pharmacological inhibition of Rev-erbα raised the possibility of an increase in neuronal cell death after the CCI challenge. To investigate if the activity of Rev-erbα could modulate the process of apoptotic cell death, brain sections from both the prefrontal cortex and hippocampal CA1 regions were prepared and immunofluorescent stained for the TUNEL (terminal deoxynucleotidyl transferase dUTP nick end labeling)-positive neurons following the CCI challenge. The results demonstrated that the CCI challenge profoundly increased the number of TUNEL-positive apoptotic neuronal cells at three days following TBI in both the prefrontal cortex (Figure 3D,F) and hippocampal CA1 regions (Figure 3E,G). Interestingly, the pharmacological inhibition of Rev-erbα leads to even higher numbers of TUNEL-positive apoptotic neurons in both the prefrontal cortex (Figure 3D,F; TBI with vehicle, 840 ± 93.3 cells per mm^2^ vs. TBI with SR8278, 1400 ± 160.6 cells per mm^2^; *p* < 0.01) and hippocampal CA1 regions (Figure 3E,G, TBI with vehicle, 760 ± 93.3 cells per mm^2^ vs. TBI with SR8278, 1160 ± 151.4 cells per mm^2^, *p* < 0.05).

Moreover, real-time PCR analyses demonstrated that the pharmacological inhibition of Rev-erbα profoundly increased the expression levels of pro-apoptotic molecules, including *Bax* and *Bak1* at three days post-CCI challenge (Figure 3J,K). No significant differences were observed for the expression levels of anti-apoptotic molecules such as *Bcl2* or *Bcl2l1* between the vehicle-injected and SR8278-injected animals (Figure 3H,I). These findings strongly suggested that Rev-erbα could play a crucial protective role in modulating apoptotic cell death and functional recovery following TBI.

### 3.4. Pharmacological Activation of Rev-Erbα Promotes Functional Recovery

**Figure 4:**
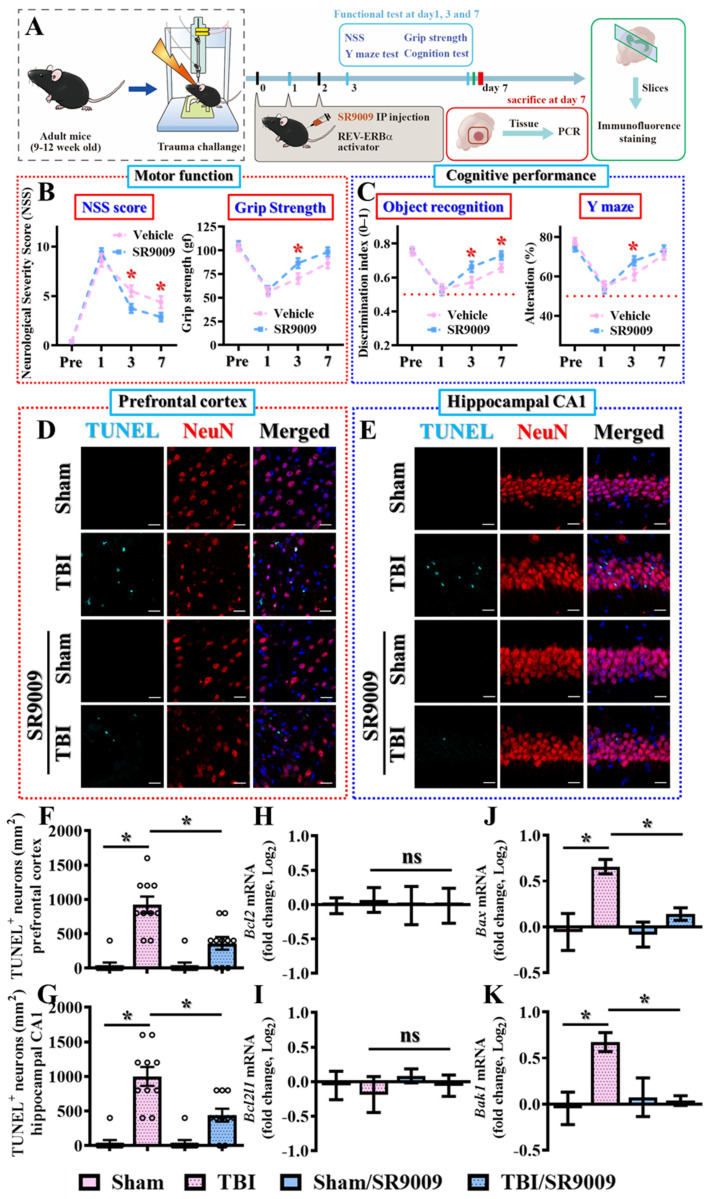
Pharmacological activation of Rev-erbα promotes functional recovery from TBI. (**A**) The illustrating image demonstrates the procedures for the experimental designs with SR9009. (**B**) Behavioral performance for the evaluation of motor function based on neurological severity scores and grip strength at different time points after TBI challenge. Data are presented as mean ± SEM. *n* = 10 mice. (**C**) Behavioral performance for the evaluation of cognitive function was evaluated based on object recognition and the Y-maze task at different time points after TBI challenge. Data are presented as mean ± SEM. *n* = 10 mice. (**D**) Representative images for the TUNEL-positive (cyan), neuron (NeuN, red) and DAPI (blue) assays in the prefrontal cortex from different experimental groups (scale = 20 μm). (**E**) Representative images for the TUNEL-positive (cyan), neuron (NeuN, red) and DAPI (blue) in the hippocampal CA1 regions from different experimental groups (scale = 20 μm). (**F**) Quantification of the number of TUNEL-positive neuronal cells in the prefrontal cortex. *n* = 10 images from 4 mice. (**G**) Quantification of the number of TUNEL-positive neuronal cells in the hippocampal CA1 regions from different experimental groups. *n* = 10 images from 4 mice. (**H**–**K**) Quantification of the fold changes in gene expression levels of *Bcl2*, *Bcl2l1*, *Bax* and *Bak1* in the hippocampus at three days post-CCI surgery. *n* = 5 mice. Data are presented as mean ± SEM and then analyzed by the one-way ANOVA test followed by Bonferroni’s post hoc analysis. * *p* < 0.05; ns represents not significant.

Having completed loss-of-function experiments with the Rev-erbα antagonist, we then wanted to investigate whether the activation of Rev-erbα could promote functional recovery from TBI. To address this, we employed the widely used pharmacological agonist SR9009, which activates Rev-erbα effectively [28,29]. Similarly, the functional activation of Rev-erbα by SR9009 was also confirmed by a significant decrease in the mRNA levels of Bmal1 which can be transcriptionally suppressed by Rev-erbα (Figure S1), without significant alterations in the mRNA levels of Nr1d1 and Per1. Experimentally, mice aged between 9 and 12 weeks were administered with 50 mg/kg SR9009 intraperitoneally at one, twenty-four, and forty-eight hours post-CCI challenge. Subsequently, motor and cognitive performances were assessed at different time points post-CCI challenge (Figure 4A). The results indicated that the pharmacological activation of Rev-erbα significantly enhanced functional recovery from traumatic brain injury, as evidenced by the lower neurological severity scores (NSS) and improved grip strength compared to the vehicle-treated group of animals (Figure 4B). Meanwhile, treatment with SR9009 also significantly improved cognitive performance, demonstrated by the higher discrimination indexes in the novel object location task and increased alternative selection in the spontaneous alternation Y-maze task (Figure 4C).

To elucidate the possible modulation of apoptotic cell death post-CCI via the activation of Rev-erbα, brain sections from both prefrontal cortex and hippocampal CA1 regions were analyzed. A comparison of immunofluorescent images of TUNEL-positive neurons between different experimental groups demonstrated that the activation of Rev-erbα via SR9009 treatment significantly reduced the number of apoptotic neurons at three days post-TBI (Figure 4D–G; prefrontal cortex; TBI with vehicle, 920 ± 120 cells per mm^2^ vs. TBI with SR9009, 360 ± 93.33 cells per mm^2^, *p* < 0.01; hippocampal CA1 region, TBI with vehicle, 1000 ± 136.6 cells per mm^2^ vs. TBI with SR9009, 440 ± 93.3 cells per mm^2^; *p* < 0.01).

In the meantime, real-time PCR analyses further revealed that temporal SR9009 treatment profoundly decreased the expression levels of the pro-apoptotic molecules *Bax* and *Bak1* at three days post-CCI challenge (Figure 4J,K), while the expression of the anti-apoptotic molecules *Bcl2* or *Bcl2l1* was not significantly altered by the same treatment (Figure 4H,I). These findings further suggest that the activation of Rev-erbα ameliorates apoptotic neuronal death while promoting functional recovery by modulating the balance between pro- and anti-apoptotic cellular events.

### 3.5. Modulation of TBI-Induced Inflammatory Responses by Rev-Erbα

Our findings indicate that alterations in Rev-erbα activity can modulate apoptotic neuronal death and influence functional recovery under the pathological conditions of traumatic brain injury. To our surprise, the mRNA expression levels of the pro-apoptotic molecules Bax and Bak1, as well as the anti-apoptotic molecules Bcl2 and Bcl2l1, were not significantly affected by either the inhibition or activation of Rev-erbα under physiological conditions, as shown by the comparisons between vehicle and drug-treated sham-operated animals (Figure 3H–K and Figure 4H–K). This suggests that Rev-erbα might indirectly modulate apoptotic neuronal death by regulating pathological events underlying TBI, such as the exaggerated inflammatory events or excessive oxidative stress.

Accumulating evidence indicates that exaggerated inflammatory events provoked by reactive microglial cells and astrocytes highly correlate with extensive neuronal cell damage and impaired functional recovery following traumatic brain injury. Reactive microglial cells and astrocytes exhibit profound morphological changes accompanied by an increased release of pro-inflammatory cytokines, contributing to secondary injury mechanisms. Since recent studies suggest that Rev-erbα plays crucial roles in modulating these inflammatory events, by regulating the activity of Rev-erbα, it might be possible to mitigate the excessive inflammation and consequent neuronal damage, thereby promoting better functional recovery following TBI.

**Figure 5:**
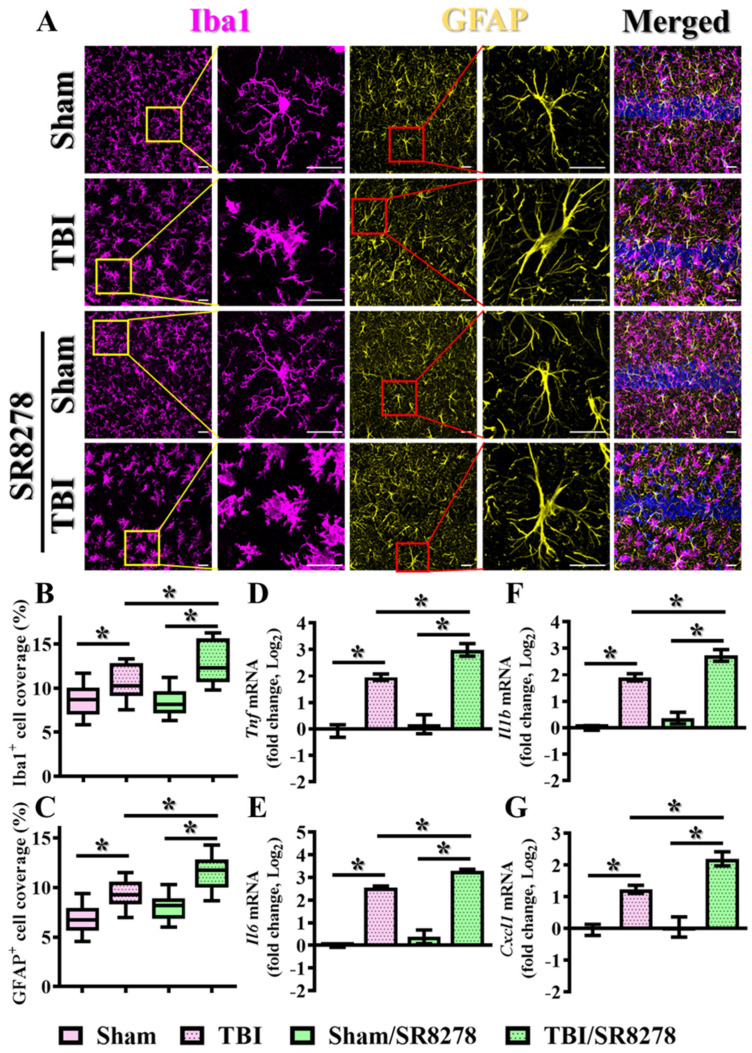
Exaggeration of TBI-induced inflammatory responses by SR8278. (**A**) Representative images showing the IBA1-positive microglial cells (magenta) with the GFAP-positive astrocytes (yellow) from different experimental groups. DAPI (blue) is used for the nuclear stain (scale = 20 μm). (**B**) Quantitative analyses of the percentage in the area of IBA1-positive cell coverage between different experimental groups. *n* = 10 images from 4 mice. (**C**) Quantitative analyses of the percentage in the area of GFAP-positive cell coverage between different experimental groups. *n* = 10 images from 4 mice. (**D**–**G**) Quantification of the fold changes in gene expression levels of *Tnf*, *Il-6*, *Il-1β*, and *Cxcl1* between different experimental groups of animals. *n* = 5 mice. Data are presented as mean ± SEM and then analyzed by the one-way ANOVA test followed by Bonferroni’s post hoc analysis. * *p* < 0.05.

To investigate the impact of Rev-erbα in the modulation of microgliosis, IBA1 (ionized calcium-binding adapter molecule 1) staining was used for the visualization of the morphological activation of microglial cells induced by the CCI challenge. This activation is characterized by the morphological transformation of microglia from an extremely ramified structure into an amoeboid figure with larger cell bodies and thicker processes. Our results indicate that undergoing the CCI insults significantly induce the morphological activation of microglial cells (Figure 5A), as evidenced by the increase in the percentage of the area covered by IBA1-positive cells (Figure 5B; sham, 8.752 ± 0.615% vs. TBI, 10.57 ± 0.6745%, *p* < 0.05). Furthermore, the pharmacological suppression of Rev-erbα by SR8278 significantly intensified the phenotypes of inflammatory microgliosis induced by the CCI challenge, as reflected by an even greater percentage of IBA1-positive area coverage post-CCI (Figure 5A,B; TBI, 10.57 ± 0.6745% vs. TBI/SR8278, 12.95 ± 0.8198%; *p* < 0.05).

At the same time, GFAP (glial fibrillary acidic protein) staining was applied for the labeling and visualization of astroglial cells. As shown in Figure 5A, the phenomenon of astrogliosis is evidenced by a substantial morphological transformation of astroglial cells, characterized by phenotypes of thicker and more highly elaborate processes following the CCI challenge (Figure 5A,C). Interestingly, our results indicated that CCI-induced astrogliosis is significantly enhanced via the pharmacological suppression of Rev-erbα by SR8278, as evidenced by the increased percentage of the area covered by GFAP-positive cells following CCI challenge (Figure 5A,C; TBI, 9.264 ± 0.4763% vs. TBI/SR8278, 11.39 ± 0.599%, *p* < 0.05). Similar results demonstrating the exaggeration of TBI-induced activation of microglia and astrocytes by SR8278 following TBI were also confirmed in the prefrontal cortex (Figure S2A,C).

Additionally, real-time PCR analyses of pro-inflammatory cytokines and chemokines such as *Tnf*, *Il6*, *Il1b*, and *Cxcl1* demonstrated that the pharmacological inhibition of Rev-erbα significantly intensified inflammatory responses at three days post-CCI challenge (Figure 5D–G). These results strongly highlight the essential role of Rev-erbα in modulating the inflammatory response following TBI.

**Figure 6:**
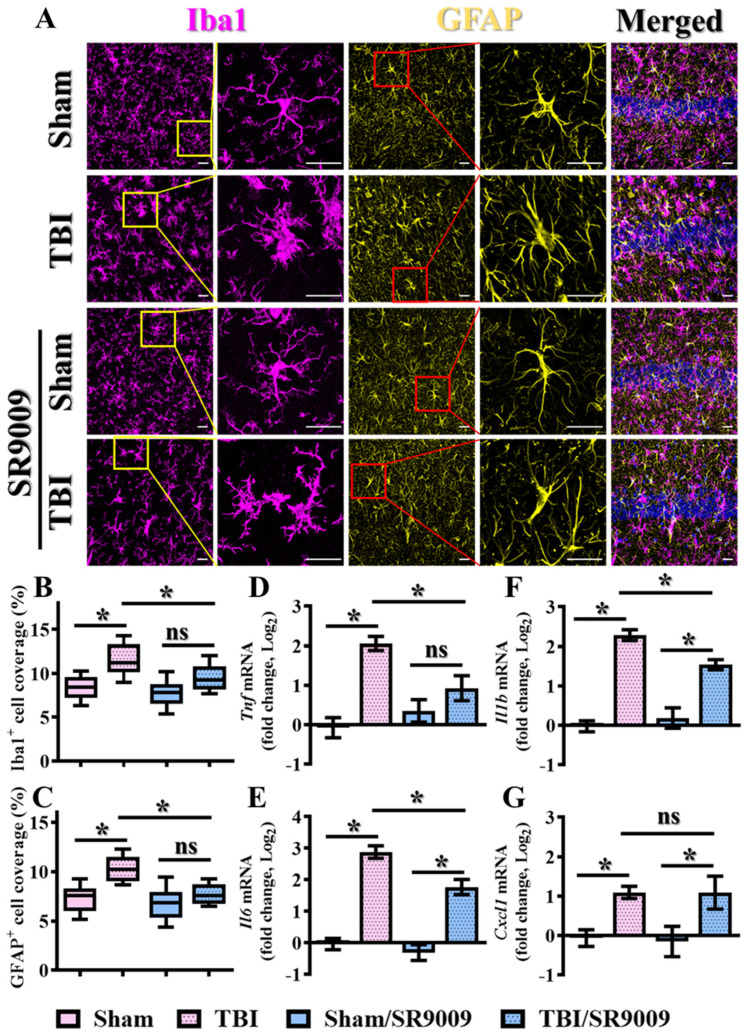
Amelioration of TBI-induced inflammatory responses by SR9009. (**A**) Representative images showing the IBA1-positive microglial cells (magenta) with the GFAP-positive astrocytes (yellow) in the hippocampal CA1 regions from different experimental groups. DAPI (blue) is used for the nuclear stain (scale = 20 μm). (**B**) Quantitative analyses of the percentage in the area of IBA1-positive cell coverage between different experimental groups. *n* = 10 images from 4 mice. (**C**) Quantitative analyses of the percentage in the area of GFAP-positive cell coverage between different experimental groups. *n* = 10 images from 4 mice. (**D**–**G**) Quantification of the fold changes in gene expression levels of different cytokines and chemokines such as *Tnf*, *Il-6*, *Il-1β*, and *Cxcl1* between different experimental groups of animals. *n* = 5 mice. Data are presented as mean ± SEM and then analyzed by the one-way ANOVA test followed by Bonferroni’s post hoc analysis. * *p* < 0.05; ns represents not significant.

To further explore the therapeutic potential of Rev-erbα activation in the modulation of TBI-induced inflammatory responses, the impact of the pharmacological activation of Rev-erbα was also evaluated. Again, adult mice aged between nine and twelve weeks received 50 mg/kg SR9009 intraperitoneally at one, twenty-four, and forty-eight hours post-CCI challenge, and brain sections were prepared at three days after CCI challenge to explore the alteration in the phenotypes of microgliosis and astrogliosis. Our results indicated that the activation of Rev-erbα via SR9009 treatment significantly reduced the morphological activation of microglial cells, as evidenced by a decrease in the percentage of the area covered by IBA1-positive cells following the CCI challenge (Figure 6A,B; TBI, 11.63 ± 0.6193% vs. TBI/SR9009, 9.507 ± 0.5103%; *p* < 0.05). Similarly, the pharmacological activation of Rev-erbα profoundly ameliorated CCI-induced astrogliosis, as reflected in the reduction in the percentage of the area covered by GFAP-positive cells (Figure 6A–C; TBI, 10.23 ± 0.4312% vs. TBI/SR9009, 7.656 ± 0.3428%; *p* < 0.01). Similar results demonstrating the amelioration of TBI-induced activation of microglia and astrocytes by SR9009 following TBI were also confirmed in the prefrontal cortex (Figure S3A–C).

Moreover, real-time PCR analyses revealed that the temporal activation of Rev-erbα via SR9009 treatment significantly decreased the expression levels of different pro-inflammatory cytokines, including *Tnf*, *Il6*, and *Il1b*, at three days post-CCI challenge (Figure 6D–G). These findings highlight the potential of Rev-erbα in the modulation of inflammatory events under both physiological and pathological conditions, which critically contribute to functional recovery following traumatic brain injury.

### 3.6. Modulation of TBI-Induced Excessive Oxidative Stress by Rev-erbα

Oxidative stress overload is a well-known contributor to the pathology of TBI, leading to cellular damage and impairing recovery [30,31,32]. ROS and RNS produced following TBI can cause lipid peroxidation, protein oxidation, and DNA damage. To understand the role of Rev-erbα in modulating oxidative stress following TBI, we utilized both the pharmacological antagonist SR8278 and the agonist SR9009. Experimentally, the levels of oxidative stress were assessed via immunofluorescent staining for 4-hydroxynonenal (4-HNE) and 8-oxoguanine (8-oxo-dG), which are markers of lipid peroxidation and DNA oxidation, respectively.

**Figure 7:**
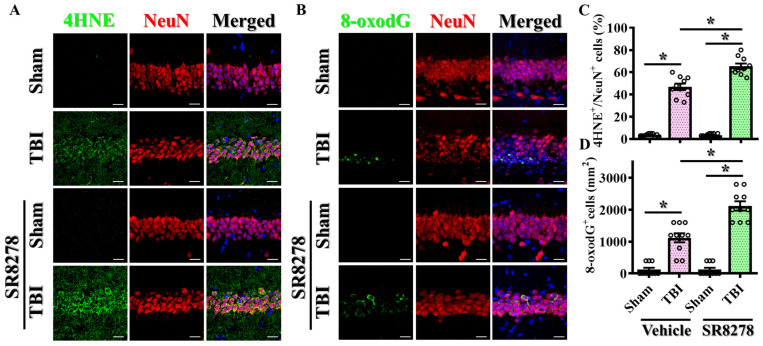
Exaggeration of TBI-induced excessive oxidative stress by SR8278. (**A**) Representative images showing the 4HNE-positive (green) neurons (NeuN, red) in the hippocampal CA1 regions from different experimental groups. DAPI (blue) is used for the nuclear stain (scale = 20 μm). (**B**) Representative images showing the 8-oxo-dG-positive (green) neurons (NeuN, red) in the hippocampal CA1 regions from different experimental groups. DAPI (blue) is used for the nuclear stain (scale = 20 μm). (**C**) Quantitative analyses of the percentage in 4HNE-positive neuronal cells between different experimental groups. *n* = 10 images from 4 mice. (**D**) Quantitative analyses of the number of 8-oxo-dG-positive neuronal cells between different experimental groups. *n* = 10 images from 4 mice. Data are presented as mean ± SEM and then analyzed by the one-way ANOVA test followed by Bonferroni’s post hoc analysis. * *p* < 0.05.

To understand the impact of the pharmacological suppression of Rev-erbα on the modulation of TBI-induced oxidative stress, brain sections from the hippocampal CA1 regions were prepared from sham-operated and CCI-challenged mice treated with 25 mg/kg SR8278. 4-hydroxynonenal (4-HNE), the well-known toxic byproduct of lipid peroxidation, has been widely recognized as a sensitive marker to reflect the levels of oxidative stress and lipid peroxidation. By immunostaining the intensity of 4-HNE adducts on proteins, our results demonstrated that the CCI challenge significantly increased the percentage of neurons with 4-HNE-positive puncta in the hippocampal CA1 regions at three days post-CCI (Figure 7A,C, TBI with vehicle, 46.9 ± 2.861% vs. sham with vehicle, 3.60 ± 0.371%, *p* < 0.001). Remarkably, the pharmacological inhibition of Rev-erbα with SR8278 further exacerbated oxidative stress, as evidenced by a significantly increased percentage of neuronal cells with 4-HNE positive puncta compared to vehicle-treated CCI mice (Figure 7A,C; TBI with SR8278, 65.3 ± 2.517% vs. TBI with vehicle, 46.9 ± 2.861%; *p* < 0.001).

In addition to the visualization of oxidative protein adducts via 4-HNE staining after the CCI challenge, oxidative DNA damage is also known to play crucial roles in the pathogenesis of various diseases, including metabolic and neurodegenerative disorders. 8-Oxo-7,8-dihydro-2′-deoxyguanosine (8-oxodG), the major form of free radical-induced oxidative lesions on nuclear and mitochondrial DNA, is widely used as the biomarker to study the levels of excessive oxidative stress. At the beginning, we first co-stained 8-oxo-dG with different markers. We found that the majority of 8-oxo-dG-positive cells were neurons at both one and three days following TBI. Therefore, in the current study, we decided to focus on investigating the impact of Rev-erbα modulation on the changes in oxidative stress in neuronal cells. Using an 8-oxodG-specific antibody co-stained with neuronal marker, NeuN, our results demonstrated that the CCI challenge significantly increased the number of neurons co-stained with 8-oxodG-positive signals in the hippocampus at three days post-CCI (Figure 7B,D; TBI with vehicle, 1120 ± 143.6 cells per mm^2^ vs. sham with vehicle, 120 ± 61.1 cells per mm^2^; *p* < 0.001).

Furthermore, the pharmacological suppression of Rev-erbα with SR8278 further increased the levels of excessive oxidative DNA damage, as reflected in the significantly increased number of neuronal cells with 8-oxodG-positive staining compared to vehicle-treated CCI mice (Figure 7B,D; TBI with SR8278, 2120 ± 146.7 cells per mm^2^ vs. TBI with vehicle, 1120 ± 143.6 cells per mm^2^; *p* < 0.001). Similar results demonstrating the exaggeration of TBI-induced excessive oxidative stress by SR8278 following TBI were also confirmed in the prefrontal cortex (Figure S4A–D). This increase in oxidative DNA damage underscores the crucial role of oxidative stress in the pathophysiology of TBI, thus highlighting the potential impact of Rev-erbα modulation on mitigating TBI-induced deleterious impacts.

**Figure 8:**
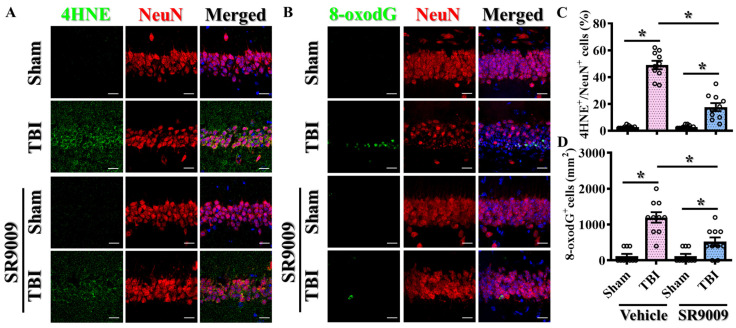
Amelioration of TBI-induced oxidative stress overload by SR9009. (**A**) Representative images showing the 4HNE-positive (green) neurons (NeuN, red) in the hippocampal CA1 regions from different experimental groups. DAPI (blue) is used for the nuclear stain (scale = 20 μm). (**B**) Representative images showing the 8-oxo-dG-positive (green) neurons (NeuN, red) from different experimental groups. DAPI (blue) is used for the nuclear stain (scale = 20 μm). (**C**) Quantitative analyses of the percentage of 4HNE-positive neuronal cells between different experimental groups. *n* = 10 images from 4 mice. (**D**) Quantitative analyses of the number of 8-oxo-dG-positive neuronal cells between different experimental groups. *n* = 10 images from 4 mice. Data are presented as mean ± SEM and then analyzed by the one-way ANOVA test followed by Bonferroni’s post hoc analysis. * *p* < 0.05.

To further explore the therapeutic potential of Rev-erbα activation in the modulation of TBI-induced excessive oxidative stress, the impact of the pharmacological activation of Rev-erbα was also evaluated. Similarly, brain sections from the hippocampal CA1 regions were prepared from sham-operated and CCI-challenged mice treated with 50 mg/kg SR9009. Through immunofluorescent staining for the labeling of 4-HNE adducts on proteins, our results demonstrated that the CCI challenge profoundly increased the percentage of neuronal cells with 4-HNE-positive puncta in the hippocampal CA1 regions at three days post-CCI (Figure 8A,C). Remarkably, the pharmacological activation of Rev-erbα with SR9009 significantly reduced oxidative stress, as evidenced by a lower percentage of neurons with 4-HNE-positive puncta compared to vehicle-treated CCI mice (Figure 8A,C; TBI with SR9009, 17.6 ± 2.971% vs. TBI with vehicle, 49.0 ± 3.13%; *p* < 0.001).

Furthermore, the pharmacological activation of Rev-erbα with SR9009 significantly reduced the levels of oxidative DNA damage, as evidenced by the decrease in the number of neurons with 8-oxodG-positive staining compared to vehicle-treated CCI mice at three days post-CCI (Figure 8B,D; TBI with SR9009, 520 ± 120 cells per mm^2^ vs. TBI with vehicle, 1200 ± 146.1 cells per mm^2^, *p* < 0.001). Similar results demonstrating the amelioration of TBI-induced excessive oxidative stress by SR9009 following TBI were also confirmed in the prefrontal cortex (Figure S5A–D). These findings highlight the critical role of Rev-erbα in modulating oxidative stress in the context of TBI and suggest that therapeutic strategies targeting Rev-erbα may help to mitigate oxidative neuronal damage and improve functional outcomes following TBI.

To further elucidate the underlying molecular mechanisms by which Rev-erbα regulates oxidative stress pathways, we then conducted real-time PCR analyses for several key molecules that are involved in oxidative stress regulation, including NRF2, Sod1, Sod2, and Catalase. Nuclear factor erythroid 2-related factor 2 (NRF2) is well known as a crucial transcription factor that regulates the expression of several antioxidant proteins and detoxifying enzymes, thus contributing to proper modulation in cellular defense against oxidative stress [33]. Superoxide dismutase 1 (SOD1) and superoxide dismutase 2 (SOD2) are critical enzymes that catalyze the dismutation of superoxide free radicals into oxygen and hydrogen peroxide, thereby reducing oxidative damage to the cells. Sod1 is primarily located in the cytoplasm, while Sod2 is found in the mitochondria, highlighting their complementary roles in the modulation of excessive oxidative stress [34]. Catalase is another essential antioxidant molecule that decomposes hydrogen peroxide into water and oxygen, avoiding its harmful oxidative damage to the cells. Together, these genes form a critical part of the cellular antioxidant defense system, mitigating oxidative damage and maintaining cellular homeostasis.

**Figure 9:**
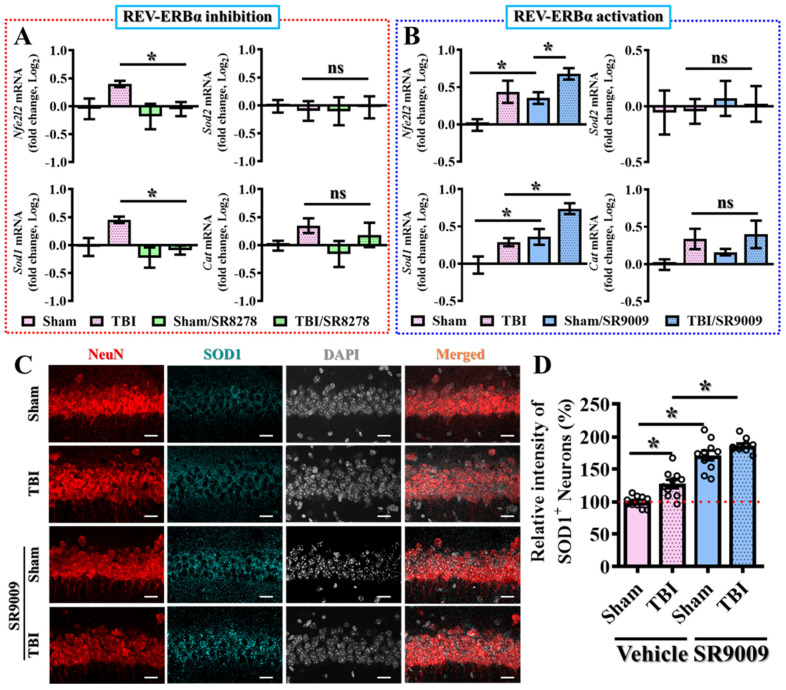
Bidirectional modulation of the molecules related to oxidative stress by Rev-erbα. (**A**) Quantitative analyses of the fold changes in gene expression levels of *Nfe2l2*, *Sod1*, *Sod2*, and *Cat* for the experiments with SR8278 application between different experimental groups. *n* = 5 mice. (**B**) Quantitative analyses of the fold changes in gene expression levels of *Nfe2l2*, *Sod1*, *Sod2*, and *Cat* for the experiments with SR9009 application between different experimental groups. *n* = 5 mice. (**C**) Representative images showing the SOD1-positive (cyan) neurons (NeuN, red) in the hippocampal CA1 regions from different experimental groups. DAPI (blue) is used for the nuclear stain (scale = 20 μm). (**D**) Quantitative analyses of the relative signal intensity of SOD1-positive neuronal cells between different experimental groups. *n* = 10 images from 4 mice. Data are presented as mean ± SEM and then analyzed by the one-way ANOVA test followed by Bonferroni’s post hoc analysis. * *p* < 0.05; ns represents not significant.

Our real-time PCR analyses demonstrated that the pharmacological inhibition or activation of Rev-erbα significantly influenced the expression of these key molecules in oxidative stress regulation at three days post-CCI challenge (Figure 9A,B). Specifically, the pharmacological inhibition of Rev-erbα significantly attenuated the mRNA levels of *Nfe2l2* (*Nrf2*) and *Sod1* compared to the vehicle-injected TBI group of animals (Figure 9A). Conversely, the pharmacological activation of Rev-erbα via SR9009 treatment profoundly increased the expression levels of *Nfe2l2* and *Sod1* at three days following the CCI challenge (Figure 9B).

Additionally, we also confirmed the protein expression levels of SOD1 via immunofluorescent staining. By preparing brain sections from sham-operated or TBI mice at three days post-CCI, we found that the pharmacological activation of Rev-erbα significantly increased the protein expression levels of SOD1 in the hippocampal CA1 regions compared to the vehicle-treated TBI group of animals (Figure 9C,D; TBI with SR9009, 186.6 ± 3.34% vs. TBI with vehicle, 127.4 ± 6.459; *p* < 0.05). More interestingly, we found that the pharmacological activation of Rev-erbα also significantly increased both the mRNA and protein expression levels of SOD1 under physiological conditions without the CCI challenge (Figure 9B–D; for *Sod1* mRNA, sham with SR9009, 131.8 ± 10.35% vs. sham with vehicle, 100 ± 7.698, *p* < 0.05; for SOD1 protein, sham with SR9009, 171.2 ± 7.598% vs. sham with vehicle, 100 ± 2.794; *p* < 0.001).

These results strongly support the crucial role of Rev-erbα in modulating the homeostatic balance of oxidative stress levels in the brain under both physiological and pathological conditions. The enhanced expression of these antioxidant defenses through Rev-erbα activation highlights its potential as a therapeutic target for mitigating the deleterious effects of TBI and maintaining oxidative homeostasis.

## 4. Discussion

Inspired by the observed sustained decrease in Rev-erbα expression after TBI challenge and the functional correlation of individual Rev-erbα levels with functional outcomes post-CCI, our study aimed to understand the critical role of the circadian protein Rev-erbα in the pathophysiology of TBI and its potential as a therapeutic target. Our results provide compelling evidence showing that both the pharmacological inhibition and activation of Rev-erbα significantly influence inflammatory responses and oxidative stress, which are crucial factors in the progression and recovery from the deleterious impacts of brain trauma.

### 4.1. Mechanisms of Rev-erbα’s Action in TBI

The mechanistic insights provided by our study indicate that Rev-erbα modulates the expression of key oxidative stress and inflammatory regulatory genes, which have long been reported as crucial for cell survival under the conditions of pathological challenge such as brain trauma. NRF2 is a critical transcription factor that regulates the expression of antioxidant enzymes, while SOD1 is essential for dismutating superoxide radicals into less harmful molecules. Accumulating evidence has demonstrated the relationship between NRF2 signaling with cell death. One key study found that TBI leads to the downregulation of transcription factor NRF2, which pathologically contributes to increased oxidative stress, neuroinflammation, and apoptotic cell death. This suggests that targeting the NRF2 pathway could be a promising therapeutic approach for mitigating the secondary injury mechanisms resulting from TBI [35,36].

In addition to oxidative stress-related genes, Rev-erbα also influences the expression of inflammatory mediators. SR9009 application leads to a significant reduction in pro-inflammatory cytokines such as TNF-α, IL-6, and IL-1β, and this downregulation of inflammatory genes likely contributes to the decreased inflammatory events and reduced neuronal cell death seen in treated mice. Interestingly, a growing body of evidence suggests crosstalk between oxidative stress and inflammatory events under pathological conditions. For example, studies in the model of lung cancer found that Nr1d1 deficiency promoted tumorigenesis by increasing NLRP3 inflammasome activation and pro-inflammatory cytokine production, potentially mediated in part through the downregulation of NRF2 [37,38,39].

The combined effect of reducing oxidative stress and inflammation via the pharmacological activation of Rev-erbα could eventually create a more favorable environment for neuronal survival. The decreases in oxidative damage and pro-inflammatory cytokines reduce the overall cellular stress and neuronal damage that eventually lead to apoptosis. This was evidenced by the reduction in TUNEL-positive cells in the hippocampus of mice treated with SR9009.

The observed bidirectional modulation of oxidative stress and inflammatory pathways by Rev-erbα highlights its critical role in maintaining cellular homeostasis following TBI. By increasing the expression of antioxidants and anti-inflammatory genes, Rev-erbα activation helps in the amelioration of damage caused by TBI and promotes neuroprotection and recovery. Thus, the mechanisms by which Rev-erbα modulates these pathways are then linked to increased cell survival and improved functional outcomes following TBI.

### 4.2. Potential Mechanisms of Rev-erbα Reduction Following TBI

TBI itself induces a complex cascade of biological responses, including oxidative stress, inflammation, and neuronal cell death. These pathological events could contribute to the observed reduction in Rev-erbα levels following CCI challenge, which in turn may exacerbate oxidative stress and inflammation, creating a loop of regulatory interactions that worsen the overall functional outcomes.

Several studies have provided insights into the mechanisms by which inflammation and oxidative stress may lead to the reduction in Rev-erbα levels after TBI. Studies involving in vitro experiments demonstrated that pro-inflammatory cytokines such as TNF-α could reduce the mRNA expression levels of Rev-erbα. Specifically, stimulation with lipopolysaccharide (LPS) activates NF-κB signaling, which subsequently reduces Rev-erbα promoter activity and expression [40]. Further corroborating evidence has shown that LPS administration in pregnant mice leads to decreased Rev-erbα expression in decidual tissue, along with the upregulation of mRNA pro-inflammatory cytokines [41].

Additional evidence from studies involving cigarette smoke (CS) exposure in mice has shown that Nr1d1 expression levels are reduced following CS exposure [42]. CS promotes the formation of excessive ROS and oxidative stress, and disrupts the antioxidant defense system, thus mirroring our findings in the model of TBI [43]. These findings suggest that increased inflammatory mediators and oxidative stress could synergistically contribute to the reduction in Rev-erbα levels, leading to a potential feedback loop with heightened oxidative damage and impaired cellular homeostasis. Such a detrimental environment promotes neuronal cell death and impairs functional recovery. Further research is essential to fully elucidate these mechanisms and confirm the therapeutic potential of targeting Rev-erbα.

### 4.3. Corroborating Evidence for Rev-erbα Agonists in Other Types of Brain Damage

Our study provides compelling evidence showing that the modulation of Rev-erbα could significantly influence neurobehavioral outcomes following brain trauma. Mice treated with SR9009 exhibited marked improvements in behavioral outcomes, while the inhibition of Rev-erbα by SR8278 significantly exacerbated behavioral deficits. These findings underscore the critical role of Rev-erbα in mitigating the adverse effects in neurobehaviors following TBI.

Similar results have been observed in other models of brain damage. For instance, the application of SR9009 reduced neurological deficit scores in mice at 24 h after middle cerebral artery occlusion (MCAO) surgery, a model of cerebral stroke [24]. Additionally, the long-term deletion of Rev-erbα has been associated with cognitive decline, characterized by a loss of working and short-term memory (Y-maze) and long-term memory (novel object recognition) [44]. Interestingly, the activation of Rev-erbα via SR9009 treatment improved motor function in MPTP-induced Parkinson’s disease mouse models [45]. These corroborating studies collectively highlight the broad neuroprotective potential of Rev-erbα activation across various types of brain injuries.

### 4.4. Targeting Rev-erbα in Susceptible Populations

Extensive studies have indicated that circadian regulation is often disrupted in elderly individuals, contributing to their increased susceptibility to TBI and poorer functional outcomes. Given the significance of Rev-erbα in circadian regulation, reductions in its expression levels could be a significant contributing factor for increased TBI susceptibility in the elderly. Interestingly, evidence has linked the reduction in Rev-erbα to various age-related conditions and impaired cellular functions. For instance, a study demonstrated that Rev-erbα levels in the retinal pigment epithelium (RPE) decline with age. Additionally, Nr1d1 and Nr1d2 lose their rhythmicity due to the slow entrainment of the circadian system in the hypothalamus of aged mice [20,46].

These findings suggest that maintaining Rev-erbα expression levels could be crucial for mitigating the severity of TBI, particularly in aged populations, where the natural decline in or disruption of Rev-erbα activity may predispose individuals to worse outcomes following brain injury. Our findings suggested that Rev-erbα-targeting therapies could be particularly beneficial for TBI-susceptible populations, such as the elderly. By restoring proper circadian function and modulating inflammatory responses, Rev-erbα activation could potentially mitigate the heightened risk and improve recovery outcomes in these vulnerable individuals.

### 4.5. Therapeutic Implications

TBI represents a significant clinical challenge due to its high incidence and the limited effectiveness of treatments, with an ongoing need for improved diagnostic and management strategies. Despite the widespread prevalence of TBI, so far, no FDA-approved therapeutics are available for reducing the underlying damage, and acute TBI treatments have not shown effectiveness in promoting recovery in large randomized, controlled trials. There is an urgent need for effective treatments, particularly for those with mild TBI who might be suffering with long-term health complications [47].

Our findings underscore the potential of targeting Rev-erbα as a therapeutic strategy for TBI. The protective effects of SR9009 in reducing inflammation, oxidative stress, and apoptotic cell death suggest that enhancing Rev-erbα activity could promote better recovery outcomes in TBI patients. SR9009 is a synthetic Rev-erbα agonist that has been developed and optimized to improve efficacy and therapeutic performance in circadian-related diseases [28]. In an experiment involving the LPS stimulation of primary astrocyte cultures, the activation of Rev-erbα via SR9009 treatment significantly decreased IL-1β and IL-6 expression [29]. Additionally, studies have demonstrated that SR9009 enhances the induction of NRF2 and its downstream target genes, which are crucial for cytoprotective responses to oxidative stress. Furthermore, SR9009 has been shown to suppress the expression of pro-inflammatory cytokines. The role of Rev-erbα agonists in the modulation of neuroinflammation and oxidative stress opens new avenues for developing circadian-based therapies to treat brain injuries, warranting further research to elucidate the molecular mechanisms and long-term benefits of Rev-erbα activation.

## 5. Conclusions

**Figure 10:**
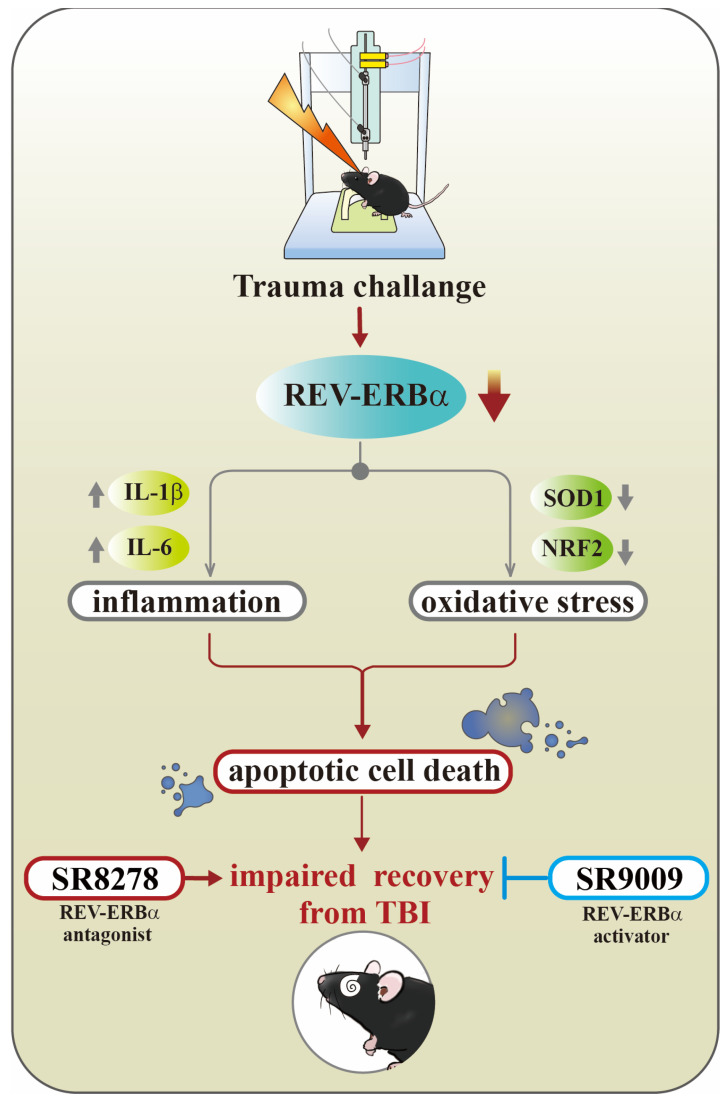
A working model summarizing the key findings of our current study. Following the challenge of traumatic brain damage, there is a significant reduction in Rev-erbα levels, a protein essential for modulating inflammatory responses and oxidative stress. The sustained dysregulation in Rev-erbα function pathologically leads to exaggerated inflammatory events and oxidative stress overload and eventually contributes to increased apoptotic cell death and affects functional recovery of the brain. Mechanistically, changes in activity of Rev-erbα modulates the expression of key oxidative stress regulatory genes such as NRF2 and SOD1, as well as the production of inflammatory mediators like TNF-α, IL-6, and IL-1β. These processes can be bidirectionally modulated by the pharmacological activator SR9009 or the inhibitor SR8278 targeting Rev-erbα. This research highlights the crucial role of circadian genes, particularly Rev-erbα, in the recovery process following brain trauma, suggesting new potential therapeutic approaches for treating brain injuries.

There is an urgent need for effective treatments for TBI. Our study reveals the critical role of the circadian protein Rev-erbα in TBI, showing that TBI downregulates Rev-erbα, leading to increased inflammation and oxidative stress. Such a regulatory loop centered on Rev-erbα exacerbates neuronal damage and hinders recovery from TBI. The pharmacological activation of Rev-erbα with SR9009 demonstrates significant potential by improving functional outcomes and reducing detrimental inflammatory and oxidative responses; conversely, its inhibition with SR8278 worsens these effects (Figure 10). In summary, these findings suggest that targeting circadian proteins could pave the way for novel and effective treatments for TBI. Future research should focus on the detailed mechanisms of Rev-erbα regulation and its application in clinical settings, particularly for TBI-susceptible groups like the elderly.

## Figures and Tables

**Table 1:** The list of the primer pairs used in the study.

Primer	Forward (Sense)	Reverse (Antisense)
**Antioxidant-Related Genes**		
*Cat*	CGGCACATGAATGGCTATGGATC	AAGCCTTCCTGCCTCTCCAACA
*Sod1*	GGTGAACCAGTTGTGTTGTCAGG	ATGAGGTCCTGCACTGGTACAG
*Sod2*	TAACGCGCAGATCATGCAGCTG	AGGCTGAAGAGCGACCTGAGTT
*Nfe2l2* (*Nrf2*)	CAGCATAGAGCAGGACATGGAG	GAACAGCGGTAGTATCAGCCAG
**Inflammatory-related genes**		
*TNF*	GGTGCCTATGTCTCAGCCTCTT	GCCATAGAACTGATGAGAGGGAG
*IL-6*	TACCACTTCACAAGTCGGAGGC	CTGCAAGTGCATCATCGTTGTTC
*IL-1β*	TGGACCTTCCAGGATGAGGACA	GTTCATCTCGGAGCCTGTAGTG
*Cxcl1*	TCCAGAGCTTGAAGGTGTTGCC	AACCAAGGGAGCTTCAGGGTCA
**Cell death-related genes**		
*Bcl-2*	CCTGTGGATGACTGAGTACCTG	AGCCAGGAGAAATCAAACAGAGG
*Bcl2l1*	GCCACCTATCTGAATGACCACC	AGGAACCAGCGGTTGAAGCGC
*Bax*	AGGATGCGTCCACCAAGAAGCT	TCCGTGTCCACGTCAGCAATCA
*Bak1*	GGAATGCCTACGAACTCTTCACC	CAAACCACGCTGGTAGACGTAC
**Circadian-related genes**		
*Per1*	GAAACCTCTGGCTGTTCCTACC	AGGCTGAAGAGGCAGTGTAGGA
*Per2*	CTGCTTGTTCCAGGCTGTGGAT	CTTCTTGTGGATGGCGAGCATC
*Nr1d1*	CAGGCTTCCGTGACCTTTCTCA	TAGGTTGTGCGGCTCAGGAACA
*Nr1d2*	CAGTGAGAAGCTGAATGCCCTC	TGCACGGATGAGTGTTTCCTGC
*Clock*	GGCTGAAAGACGGCGAGAACTT	GTGCTTCCTTGAGACTCACTGTG
*Bmal1*	ACCTCGCAGAATGTCACAGGCA	CTGAACCATCGACTTCGTAGCG
**Internal controls**		
*Gapdh*	CATCACTGCCACCCAGAAGACTG	ATGCCAGTGAGCTTCCCGTTCAG

## Data Availability

All data are available on request from the corresponding author.

## Supplementary Materials

The following supporting information can be downloaded at: https://www.mdpi.com/article/10.3390/antiox13080901/s1, Figure S1. Impact of either Rev-erbα antagonist or agonist on the expression levels of different circadian genes. Figure S2. Exaggeration of TBI-induced activation of microglia and astrocytes in the prefrontal cortex by SR8278. Figure S3. Amelioration of TBI-induced activation of microglia and astrocytes in the prefrontal cortex by SR9009. Figure S4. Exaggeration of TBI-induced excessive oxidative stress in the prefrontal cortex by SR8278. Figure S5. Amelioration of TBI-induced excessive oxidative stress in the prefrontal cortex by SR9009.
