# Supplementary material for: Targeting Circadian Protein Rev-erbα to Alleviate Inflammation, Oxidative Stress, and Enhance Functional Recovery Following Brain Trauma
Source: Antioxidants (Basel). 2024 Jul 25;13(8):901. doi: 10.3390/antiox13080901 (PMC11351136; doi:10.3390/antiox13080901)
Supplement: Supplementary file 1 [file antioxidants-13-00901-s001.zip › antioxidants-3090594-supplementary.pdf]

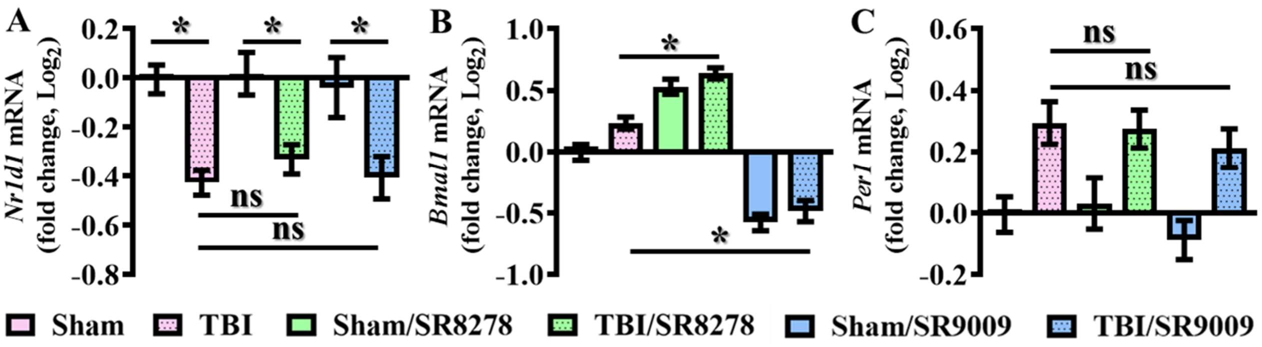

Figure S1. Impact of either Rev-erbα antagonist or agonist on the expression levels of different circadian genes. (A) Quantification of the fold changes in the gene expression levels of *Nr1D1* between different experimental groups. (B) Quantification of the fold changes in the gene expression levels of *Bmal1* between different experimental groups. (C) Quantification of the fold changes in the gene expression levels of *Per1* between different experimental groups. $n = 5$ mice. All the results were presented as mean ± SEM and analyzed by a one-way ANOVA followed by Bonferroni's post hoc analysis. * $p < 0.05$; ns represents not significant.

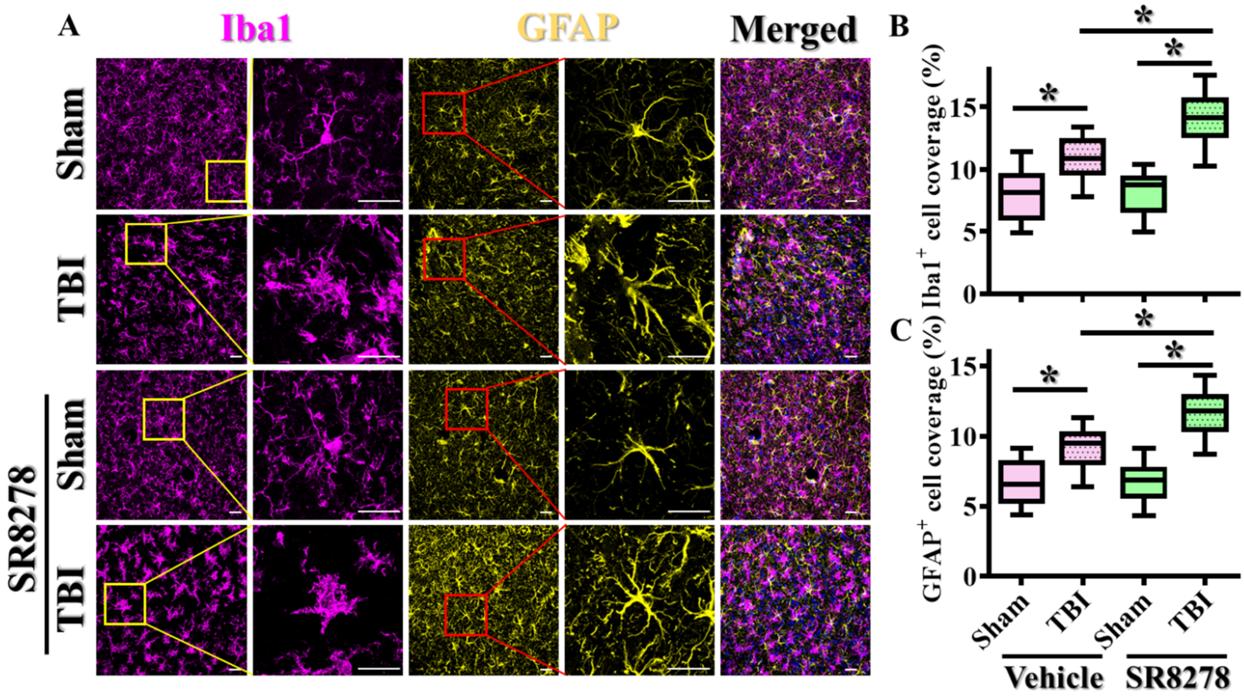

Figure S2. Exaggeration of TBI-induced activation of microglia and astrocytes in the prefrontal cortex by SR8278. (A) Representative images showing the IBA1-positive microglial cells (magenta) with the GFAP-positive astrocytes (yellow) from different experimental groups. DAPI (blue) is used for the nuclear stain (scale = 20 μm). (B) Quantitative analyses of the percentage in the area of IBA1-positive cells coverage between different experimental groups. $n = 10$ images from 4 mice. (C) Quantitative analyses of the percentage in the area of GFAP-positive cells coverage between different experimental groups. $n = 10$ images from 4 mice. Data were presented as mean ± SEM and then analyzed by the one-way ANOVA test followed by Bonferroni's post hoc analysis. * $p < 0.05$.

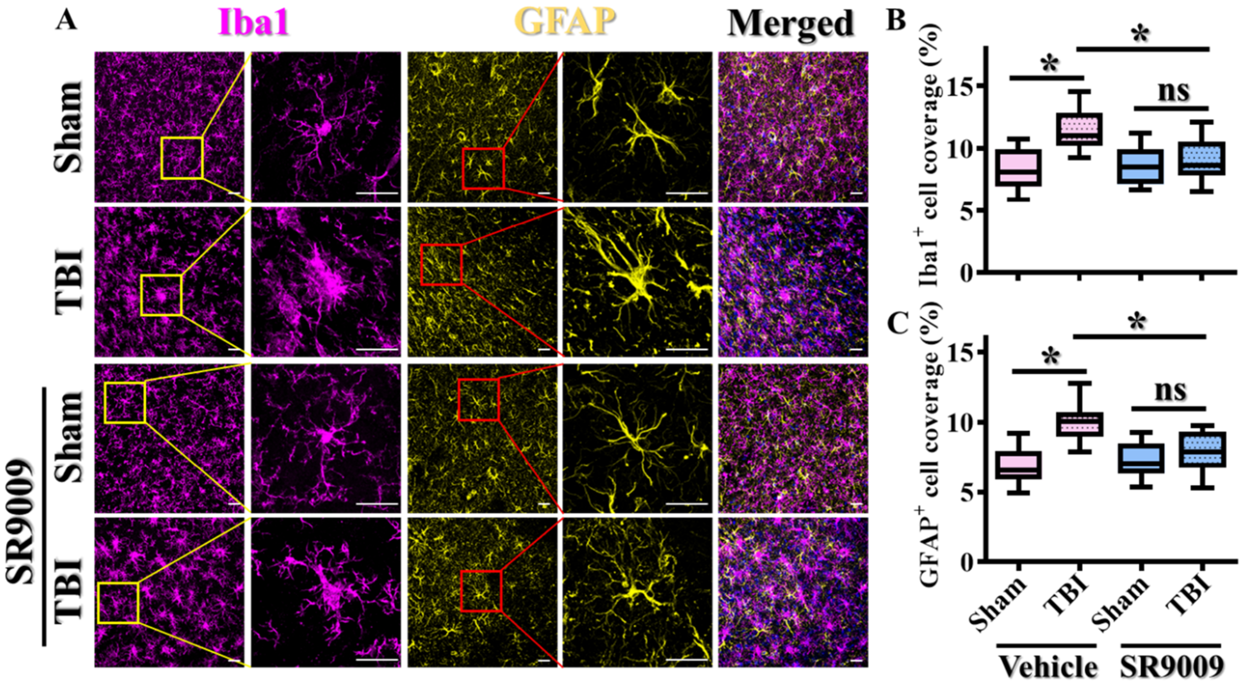

Figure S3. Ameliorationof TBI-induced activation of microglia and astrocytes in the prefrontal cortex by SR9009. (A) Representative images showing the IBA1-positive microglial cells (magenta) with the GFAP-positive astrocytes (yellow) from different experimental groups. DAPI (blue) is used for the nuclear stain (scale = 20 μm). (B) Quantitative analyses of the percentage in the area of IBA1-positive cells coverage between different experimental groups. *n* = 10 images from 4 mice. (C) Quantitative analyses of the percentage in the area of GFAP-positive cells coverage between different experimental groups. *n* = 10 images from 4 mice. Data were presented as mean ± SEM and then analyzed by the one-way ANOVA test followed by Bonferroni's post hoc analysis. * $p < 0.05$.

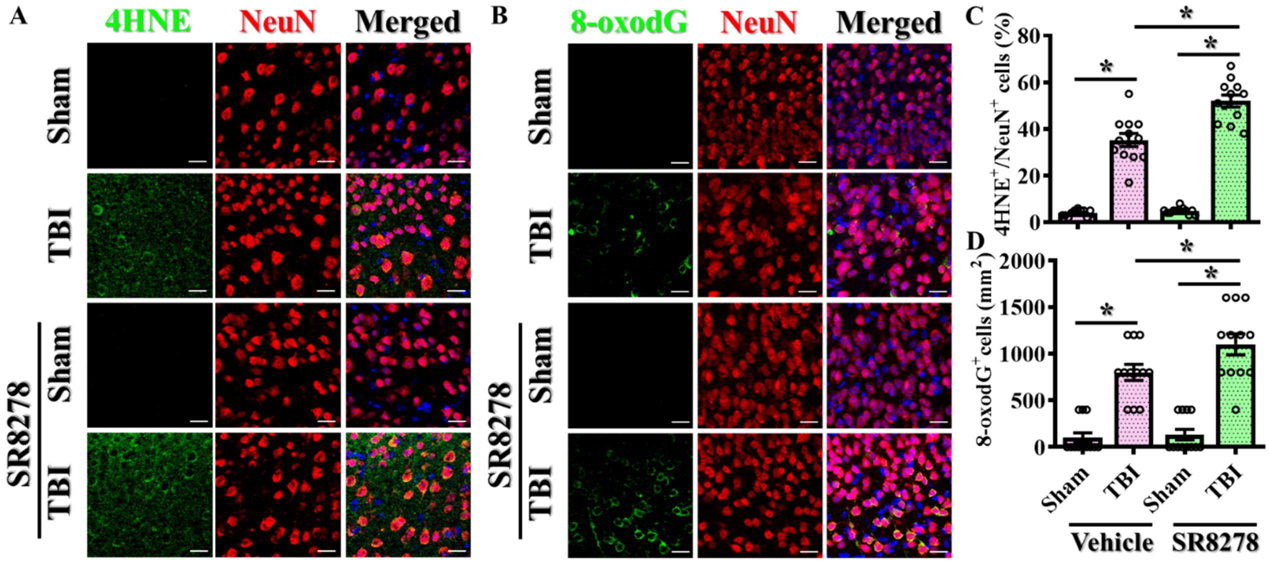

Figure S4. Exaggeration of TBI-induced excessive oxidative stress in the prefrontal cortex by SR8278. (A) Representative images showing the 4HNE-positive(green) neurons (NeuN, red) in the prefrontal cortex from different experimental groups. DAPI (blue) is used for the nuclear stain (scale = 20 μm). (B) Representative images showing the 8-oxo-dG-positive (green) neurons (NeuN, red) in the prefrontal cortex from different experimental groups. (C) Quantitative analyses of the percentage in 4HNE-positiveneuronal cells between different experimental groups. *n* = 10 images from 4 mice. (D) Quantitative analyses of the number of 8-oxo-dG-positive neuronal cells between different experimental groups. *n* = 10 images from 4 mice. Data were presented as mean ± SEM and then analyzed by the one-way ANOVA test followed by Bonferroni's post hoc analysis. * $p < 0.05$.

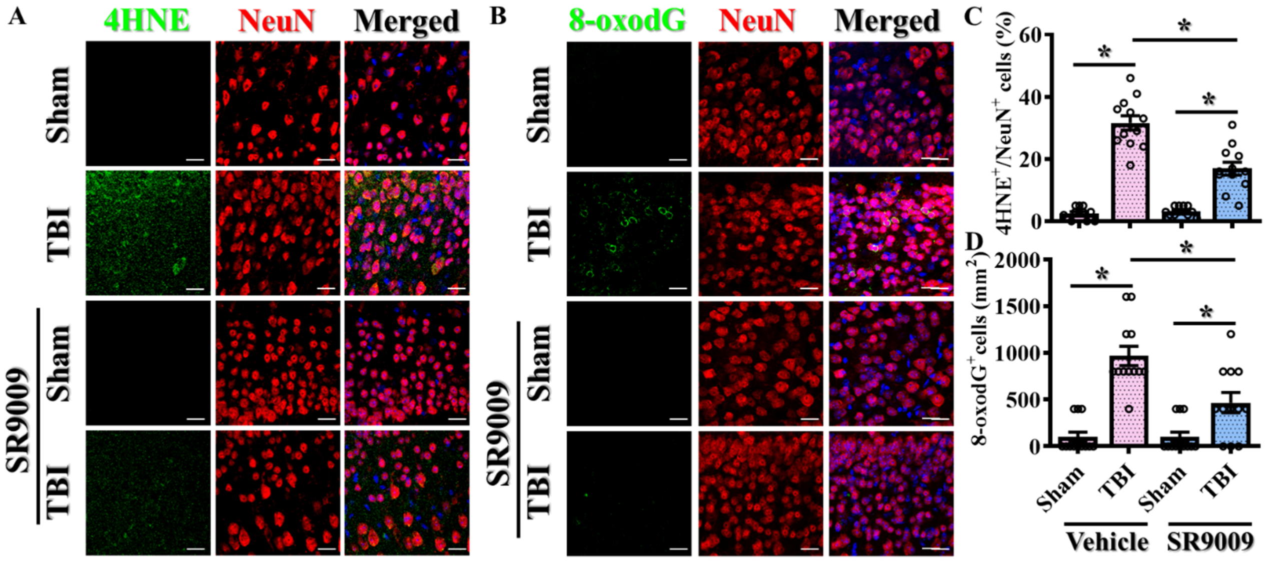

Figure S5. Amelioration of TBI-induced excessive oxidative stress in the prefrontal cortex by SR9009. (A) Representative images showing the 4HNE-positive (green) neurons (NeuN, red) in the prefrontal cortex from different experimental groups. DAPI (blue) is used for the nuclear stain (scale = 20 μm). (B) Representative images showing the 8-oxo-dG-positive (green) neurons (NeuN, red) in the prefrontal cortex from different experimental groups. (C) Quantitative analyses of the percentage in 4HNE-positive neuronal cells between different experimental groups. *n* = 10 images from 4 mice. (D) Quantitative analyses of the number of 8-oxo-dG-positive neuronal cells between different experimental groups. *n* = 10 images from 4 mice. Data were presented as mean ± SEM and then analyzed by the one-way ANOVA test followed by Bonferroni's post hoc analysis. * $p < 0.05$.
